# EffUnet-SpaGen: An Efficient and Spatial Generative Approach to Glaucoma Detection

**DOI:** 10.3390/jimaging7060092

**Published:** 2021-05-30

**Authors:** Venkatesh Krishna Adithya, Bryan M. Williams, Silvester Czanner, Srinivasan Kavitha, David S. Friedman, Colin E. Willoughby, Rengaraj Venkatesh, Gabriela Czanner

**Affiliations:** 1 Department of Glaucoma, Aravind Eye Care System, Thavalakuppam, Pondicherry 605007, India; krishnavadithya@gmail.com (V.K.A.); kavitha@aravind.org (S.K.); venkatesh@aravind.org (R.V.); 2 School of Computing and Communications, Lancaster University, Bailrigg, Lancaster LA1 4WA, UK; b.williams6@lancaster.ac.uk; 3 School of Computer Science and Mathematics, Liverpool John Moores University, Liverpool L3 3AF, UK; s.czanner@ljmu.ac.uk; 4 Glaucoma Center of Excellence, Harvard Medical School, Boston, MA 02114, USA; David_Friedman@MEEI.HARVARD.EDU; 5 Biomedical Research Institute, Ulster University, Coleraine, Co. Londonderry BT52 1SA, UK; c.willoughby@ulster.ac.uk

**Keywords:** glaucoma, diagnosis, generative model, machine learning, classification

## Abstract

Current research in automated disease detection focuses on making algorithms “slimmer” reducing the need for large training datasets and accelerating recalibration for new data while achieving high accuracy. The development of slimmer models has become a hot research topic in medical imaging. In this work, we develop a two-phase model for glaucoma detection, identifying and exploiting a redundancy in fundus image data relating particularly to the geometry. We propose a novel algorithm for the cup and disc segmentation “EffUnet” with an efficient convolution block and combine this with an extended spatial generative approach for geometry modelling and classification, termed “SpaGen” We demonstrate the high accuracy achievable by EffUnet in detecting the optic disc and cup boundaries and show how our algorithm can be quickly trained with new data by recalibrating the EffUnet layer only. Our resulting glaucoma detection algorithm, “EffUnet-SpaGen”, is optimized to significantly reduce the computational burden while at the same time surpassing the current state-of-art in glaucoma detection algorithms with AUROC 0.997 and 0.969 in the benchmark online datasets ORIGA and DRISHTI, respectively. Our algorithm also allows deformed areas of the optic rim to be displayed and investigated, providing explainability, which is crucial to successful adoption and implementation in clinical settings.

## 1. Introduction

Glaucoma is a neurodegenerative disease resulting in progressive optic nerve damage with a characteristic pattern of optic nerve damage and visual field loss. Late diagnosis is a major risk factor for permanent visual loss [1], and early glaucoma detection is key to preventing avoidable blindness. Detection of structural changes to the optic nerve using imaging or clinical examination is central to diagnosis but challenging even for highly skilled specialists. Patients can be misclassified, which is a significant challenge, especially in low-resource settings where access to clinical expertise and specialist diagnostic equipment is limited. A low-cost and accurate automated method of quantifying glaucomatous structural changes would help meet this need [2].

**Figure 1:**
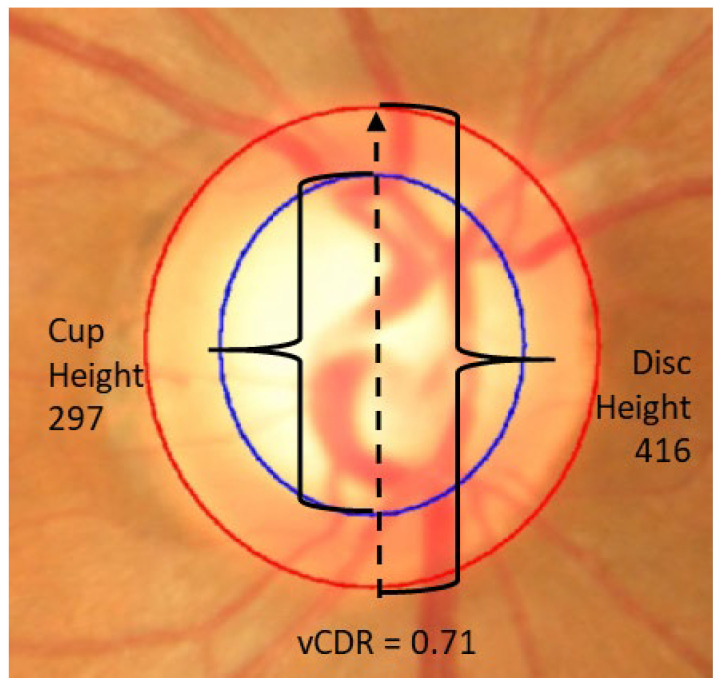
Colour fundus photograph of optic disc with two features: disc (red) and cup (blue).

A significant challenge of developing automated glaucoma detection algorithms is that a vast number of labelled colour fundus images is required for training (Figure 1). Current algorithms are very promising and show high accuracy; however, they are computationally very complex, which requires strong computing infrastructure as well as large datasets for training, for example 30 thousand images to achieve an AUROC of 0.996 [3]. Such computationally complex algorithms are challenging to implement on mobile devices for community and particularly rural disease screening, necessitating the investigation of further solutions. The access to a large amount of good quality annotated data for training is a persistent challenge, due in part to the complexity of the diagnosis. Therefore, an automated detection system that is computationally flexible to require less computing power and also requires fewer training images is a fundamental requirement.

In our paper, we present a new machine learning and generative model-based method that is able to discriminate between glaucomatous and healthy patients from standard fundus images of the optic nerve head. The proposed method revisits the convolution layers [4] and improves the generative statistical model [5]. The contribution of our work is as follows: (1) We propose a novel two-step algorithm for glaucoma detection, which traces the boundaries of the optic cup and disc efficiently, facilitating the extraction of the whole cup-to-disc profile and allowing presentation of this to the clinician for further inspection if desired, and provides an accurate glaucoma diagnosis; (2) We propose EffUnet, which is an efficient U-shaped convolutional neural network for efficient segmentation of the cup and disc; (3) To detect glaucoma, we propose a refined and extended spatial statistical generative model SpaGen, which takes into account the extracted profile and the cup to disc area ratio to improve detection; (4) We demonstrate the performance of our algorithm on two large publicly available datasets and show how it can be quickly recalibrated for independent data, by recalibrating the EffUnet layer only.

### 1.1. Background

Glaucoma is still diagnosed manually in clinical practice. Research into automated glaucoma diagnosis from fundus photographs is showing promising results. There are two main approaches to automated glaucoma detection from fundus photographs [6]. One approach involves initially automatically detecting the boundaries of the cup and disc using automated segmentation [7], which allows for the cup and disc boundaries to be used for glaucoma classification. See [8,9,10] for reviews and a recent approach in [5]. The alternative artificial intelligence (AI) approach to automated glaucoma diagnosis uses direct Deep Learning (DL) [3]. While this has clear benefits of achieving good results while obviating the necessity for explicit automated cup and disc segmentation, such approaches are trained to use all information in fundus images to differentiate glaucoma patients from those without glaucoma (see review in [11]), much of which may be redundant. These approaches require large numbers of expert-labelled images, can be more difficult to translate to new devices and are typically not explainable. The large number of the expert-labelled images is a still a problem in glaucoma due the complexity of the gold standard definition of glaucoma. To remedy the problem of large number of images, there are other approaches such as transfer learning. To solve the lack of inherent explainability, there is current research that investigates computational approaches to bring explainability to the algorithms.

A current focus is to make AI glaucoma detection algorithms “slim” in order to allow for wider use (including in low-resource settings) while also requiring fewer labelled images for training. One approach to achieve this is in realizing the redundancy in retinal fundus images for disease recognition and using this knowledge to develop lean algorithms. For example, attention maps from simple eye-tracking experiments from glaucoma grading were successfully used to improve automated glaucoma detection via an attention-based convolutional network (AG-CNN) approach [4]. However, this method requires additional data on attention maps.

Another approach to redundancy is in recognizing that the boundaries of the cup and disc in healthy eyes are similar to ellipses, and hence, a deviation from the ellipse can be utilized for discrimination [5]. Using this approach, the fundus image is reduced to a cup-to-disc profile vector of 24 numbers, and a generative model is used for classification. However, this approach uses a computationally complex DL algorithm for cup and disc segmentation. One AI approach using slimmer algorithms is to create models that are easy to calibrate on new datasets. One such approach was used in detecting diabetic retinopathy [12]; the researchers used a two-step architecture. The first step was an automated segmentation, and the second step was a disease discrimination algorithm. Using this approach, the authors showed that, for new datasets, one needs to recalibrate the segmentation algorithm while the discrimination algorithm does not change, making the computation slimmer. This approach, however, still requires a computationally intensive DL method for discrimination.

#### 1.1.1. Existing Segmentation Methods

U-Net is a U-shaped convolutional network that was originally developed for biomedical image segmentation [13]. It is composed of a down-sampling encoder layer and an up-sampling decoder layer. The encoder consists of repeated groups of two convolution layers followed by a ReLU activation function and max pooling to produce a set of encoder feature maps. The decoder path also consists of convolution layers to output decoder feature maps. Skip connections transfer the corresponding feature maps from the encoder path and concatenate them to the upsampled decoder path.

Recently, there have been various adaptations of Unet. Mnet [14] is a convolution neural network with a multi-scale input layer and a multi-scale output layer. TernausNet [15] uses a pretrained VGG model as an encoder section of Unet. LinkNet [16] exploits ResNet-18 as an encoder and also used residual blocks instead of concatenation. In [7], a pretrained ResNet-34 is used as an encoder. However, most of these models are heavy and computationally expensive. There have also been several recent attempts to segment the optic cup and disc using deep learning-based approaches, including Unet [17] and a modified Mnet with bidirectional convolutional LSTM [18]. Some methods have also aimed to deliver models with lower memory requirements. Other methods [19] proposed a modified Unet with a novel augmentation based on contrast variations, and [20] proposed CDED-Net, a computationally less expensive encoder-decoder approach with feature re-use, allowing a shallower structure to be employed.

#### 1.1.2. Generative Spatial Generative Model

Generative models are commonly used in statistics and are also known as predictive models. The idea is to fit a model and to use the model for prediction or interpolation. This is a common paradigm in statistics for longitudinal data [21,22].

In computer vision, statistical generative models are less frequently used, though their value is now being studied. For example, one group introduced a probabilistic generative layer to their convolutional neural network, and on standard benchmarks, they required 300-fold less training data while achieving similar accuracy [23].

In glaucoma detection, one group published an algorithm that uses a generative model layer for classification after a DL algorithm is used for the segmentation of the cup and disc [5]. This approach required a dataset 100-times smaller for training and achieved similar accuracy of 0.996 in internal validation. The algorithm is, however, computationally expensive due to requiring a large DL network.

## 2. Materials and Methods

Our automated supervised classification of glaucoma from fundus images aims to be computationally lean to allow wide-spread use, and to allow simple calibration on new datasets. In this section, our methods are described.

### 2.1. Our Framework

**Figure 2:**
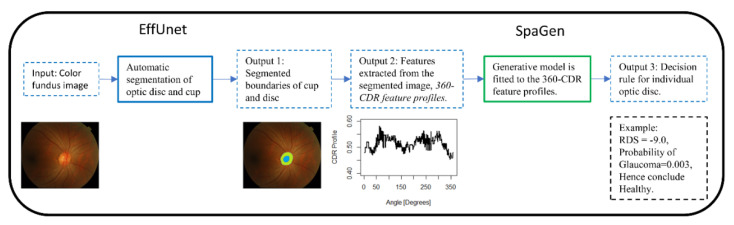
Framework of our EffUnet-SpaGen network. EffUnet is explained in Figure 3 and Figure 4, SpaGen is explained in Figure 5.

We propose a generative AI algorithm in a two-stage architecture (Figure 2). Firstly, automated segmentation of the optic cup and disc via EffUnet is performed to extract the boundaries of the cup and disc (see Output 1, Figure 2). Then, SpaGen algorithm [5] is updated by using two parameters for the variance of noise (rather than one) and by introducing the cup-to-disc area ratio (CDAR). The two variance parameters reflect the fact that variability in glaucoma images is larger than in normal images. The CDAR is added to reflect the observations of clinicians. The boundaries of the cup and disc are then used to calculate the cup-to-disc ratio (CDR) values in 24 directions at 15-degree intervals (0, 15, 30…360 degrees; see Output 2 in Figure 2). These 24 CDR values, as well as the CDAR, are then input to a spatial generative model, SpaGen. Finally, classification is carried out for each eye and output as a probability of glaucoma (see Output 3, Figure 2).

### 2.2. Segmentation of Cup and Disc via EffUnet

**Figure 3:**
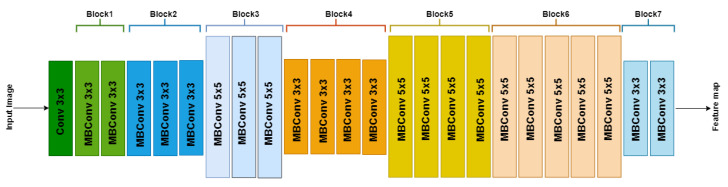
Architecture of EfficientNetB1 with MBConv as basic building blocks. The overall architecture can be divided into seven blocks, as shown. Each MBConvX block is shown with the corresponding filter size.

**Figure 4:**
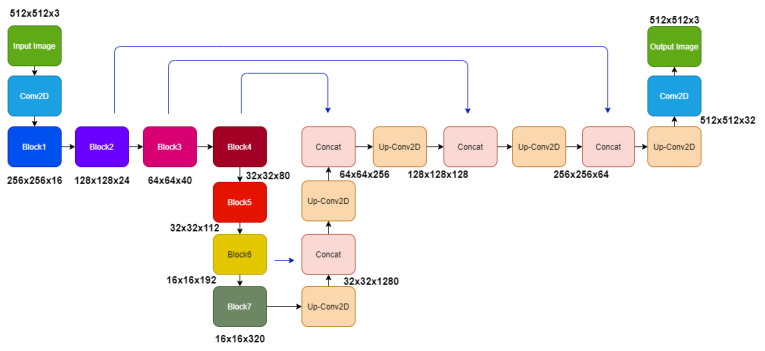
Framework of our EffUnet model. The details of Block 1–7 are shown in Figure 3. The output image (green rectangle on the right) is Output 1 in the whole architecture shown in Figure 2.

We developed EffUnet as a U-shaped convolution network with a pre-trained efficient net-B1 [24] as the encoder. This is a modification of U-Net as the main body in our deep network (Figure 3 and Figure 4).

In our modified U-Net architecture, we employ the EfficientNet-B1 as the downsampling encoder section of the U-Net architecture, while the decoder section is similar to the original U-Net architecture. EfficientNet’s main building block is a mobile inverted bottleneck MBConv [24,25], to which squeeze-and-excitation optimization [26] is also added.

To use EfficientNet-B1, the upsampling network has decoder blocks, and each decoder block is composed of a 2 × 2 upsampling 2D convolution of the previous layer output with a stride of 2, concatenated corresponding feature maps from the encoder section. The concatenated tensor is then passed through two convolution layers with ReLU activation and batch normalized before passing to the next decoder block. The final layer of the architecture is convolution with softmax with a channel number the same as the target classes and output image size the same as the input image.

Most existing segmentation models for cup and disc segmentation use a two-step process; disc segmentation to crop the region of interest and then multi-label segmentation to segment both cup and disk. Our model is applied to the entire image with just the black boundaries removed and resized to 512 × 512. Our EffUnet model is computationally less expensive with 12.6 M parameters, hence 1.9× fewer parameters than ResNet34-Unet [7], which has 24.4 M parameters. Our model converges a lot faster than the other models compared in Table 1.

### 2.3. Classification of Images via SpaGen

**Figure 5:**
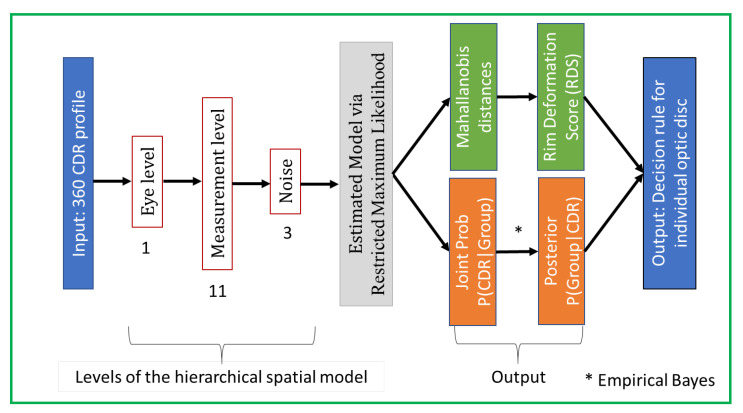
Framework of our SpaGen Model, with 15 parameters (1 + 11 + 3). This constitutes the second stage of the whole architecture (see Figure 2).

We present here an improved generative spatial algorithm (Figure 5) for disease discrimination from the shape of the cup and disc of [5]. The key novelty is in allowing for different noise modelling in disease groups and the incorporation of the cup-to-disc-area ratio (CDAR; Figure 5), which is a significant factor in detecting glaucoma [27], not previously used in an automated model. This is accomplished by including two additional parameters: one for the noise component ($\sigma_{G}^{2}$) and one for the fixed component (see $\beta_{CDAR}$). Then the final improved spatial model is a hierarchical model

(1) $$\begin{array}{c} Y_{i,d}=\beta_{0}+\beta_{G,0}I_{G}+\beta_{CDAR}CDAR\\ +\beta_{G,1}\sin\left(2\pi d/24\right)I_{G,d}+\beta_{G,1}\cos\left(2\pi d/24\right)I_{G,d}\\ +\beta_{G,3}\sin\left(4\pi d/24\right)I_{G,d}+\beta_{G,3}\cos\left(4\pi d/24\right)I_{G,d}\\ +\beta_{H,1}\sin\left(2\pi d/24\right)I_{H,d}+\beta_{H,1}\cos\left(2\pi d/24\right)I_{H,d}\\ +\beta_{H,3}\sin\left(4\pi d/24\right)I_{H,d}+\beta_{H,3}\cos\left(4\pi d/24\right)I_{H,d}\\ +z_{i}+e_{i,d} \end{array}$$

where $Y_{i,d}$ is CDR value of ith eye in dth direction (d=1,…,24); $I_{G}$ and $I_{H}$ are the indicator functions for glaucoma and healthy; $I_{G,d}$ and $I_{H,d}$ are interaction terms. The term $z_{i}$ is a random effect for of ith eye allowing to account for differences between eyes, $e_{i,d}$ is the random term accounting for random variations within the eye. The joint probability distribution of random effect and random terms is

(2) $$\left[\begin{array}{c} z_{i}\\ e_{i} \end{array}\right]\sim N\left(\left[\begin{array}{c} 0\\ 0 \end{array}\right],\;\left[\begin{array}{cc} \sigma_{z}^{2} & 0\\ 0 & V_{e} \end{array}\right]\right),$$

where $V_{e}$ is a 24×24 variance–covariance matrix of error term. We allow this matrix to be different for glaucomatous and healthy groups:

(3) $$\begin{array}{c} V_{e}=\sigma_{G}^{2}I_{24\times 24}\;\mathrm{in}\;\mathrm{glaucomatous}\;\mathrm{eye}\\ V_{e}=\sigma_{H}^{2}I_{24\times 24}\;\mathrm{in}\;\mathrm{healthy}\;\mathrm{eye}. \end{array}$$

Then, assuming the prior probabilities of the diagnostic groups glaucomatous and healthy, $p_{G}$ and $p_{H}$, and applying Bayes theorem, the posterior probability that a new eye with the observed profile vector $Y_{new}$ of 24 values of CDR (pCDR) is glaucomatous:

(4) $$p_{new,G}=\frac{p_{G}f_{G}\left(Y_{new}\;|\;\beta ,V\right)}{p_{G}f_{G}\left(Y_{new}\;|\;\beta ,V\right)+p_{H}f_{H}\left(Y_{new}\;|\;\beta ,V\right)},$$

The posterior probability in Equation (4) can be used to propose a glaucoma detection rule. The simplest detection rule is to compare this posterior probability with a predefined probability threshold, $p_{th}$:

(5) $$\begin{array}{c} \mathrm{if}\;p_{new,G}\geq p_{th},\mathrm{conclude}\;\mathrm{that}\;\mathrm{the}\;\mathrm{eye}\;\mathrm{is}\;\mathrm{glaucomatous}\\ \mathrm{if}\;p_{new,G}<p_{th},\;\mathrm{conclude}\;\mathrm{that}\;\mathrm{the}\;\mathrm{eye}\;\mathrm{is}\;\mathrm{healthy}. \end{array}$$

The probabilities have the following property

(6) $$\log\left(\frac{p_{new,G}}{1-p_{new,G}}\frac{1-p_{G}}{p_{G}}\;\right)=\frac{1}{2}\left[d_{M}\left(Y_{new},\mu_{H}\right)-d_{M}\left(Y_{new},\mu_{G}\right)\right]$$

where $d_{M}\left(Y_{i},\mu_{H}\right)$ and $d_{M}\left(Y_{i},\mu_{G}\right)$ is the Mahalanobis distance [28] of the observed data of patient i from the Healthy and Glaucomatous groups, respectively.

We then define the Rim Deformation Score (RDS) as

(7) $$RDS=\frac{1}{2}\left[d_{M}\left(Y_{new},\mu_{H}\right)-d_{M}\left(Y_{new},\mu_{G}\right)\right]$$

and this can be compared to a predefined threshold, $RDS_{th}$ to yield an equivalent decision rule

(8) $$\begin{array}{c} \mathrm{if}\;RDS_{new,G}\geq RDS_{th},\;\mathrm{conclude}\;\mathrm{that}\;\mathrm{the}\;\mathrm{eye}\;\mathrm{is}\;\mathrm{glaucomatous}\\ \mathrm{if}\;RDS_{new,G}<RDS_{th},\;\mathrm{conclude}\;\mathrm{that}\;\mathrm{the}\;\mathrm{eye}\;\mathrm{is}\;\mathrm{healthy} \end{array}$$

### 2.4. Experiments

We carried out internal validation of the performance of our EffUnet-SpaGen method in glaucoma detection on the ORIGA and DRISHTI datasets.

The ORIGA dataset is a subset of the data from the Singapore Malay Eye Study (SiMES), collected from 2004 to 2007 by the Singapore Eye Research Institute and funded by the National Medical Research Council. All images were anonymized before release. The ORIGA dataset comprises 482 healthy and 168 glaucoma images from Malay adults aged 40–80. The 650 images with manually labelled optic masks are divided into 325 training images (including 72 glaucoma cases), called ORIGA-A, and 325 testing images (including 95 glaucoma cases), called ORIGA-B [29]. The images were manually annotated by an ophthalmologist clicking on several locations of the image to indicate the optic disc and optic rim, then a best-fitting ellipse was calculated automatically. We refer to this segmentation as the ground truth. Four graders also graded the image, and a fifth grader was used for consensus.

The DRISHTI dataset [30], called DRISTHI-GS1 by the authors and referred to here as DRISHTI, is a dataset collected and annotated by Aravind Eye Hospital, Madurai, India. All 101 images are provided with segmentation ground truth. Altogether, the set contains 70 Asian glaucomatous eyes. Selected patients were 40–80 years old. DRISHTI is split into 50 training images, called DRISHTI-A, and 51 testing images, called DRISHTI-B.

For the glaucoma classification threshold, we choose a mathematically optimal threshold, which is the one that gives the closest point in the receiver operating characteristic curve (ROC) to the top left corner, where the ROC is derived from the training dataset. We used the following criteria for accuracy: area under receiver operating characteristic curve (AUROC), sensitivity, specificity, negative predictive value (NPV) and positive predictive value (PPV). We used a division of the 650 images of ORIGA into two sets, A and B, as recommended [29].

All experiments were run on a desktop computer with intel i7,16 GB RAM and a Nvidia RTX 2080 GPU (Nvidia Corporation, Santa Clara, CA, USA), which was used to train the CNN. We trained the segmentation model for 200 epochs, and the model with the best accuracy on the test set was used for evaluation. Training time for segmentation is provided in Table 1. We trained the SpaGen model by maximizing the likelihood, which has a global maximum due normal distribution of errors. The training time was 7 s.

## 3. Results

### 3.1. Segmentation Model: Computational Complexity and Accuracy

We used ORIGA’s training and testing datasets (325 images, see Experiments). For each image, black boundaries were removed, and the images were resized to 512 × 512. The performance of the proposed method EffUnet for segmenting the optic disc and optic cup was compared to the ground truth and evaluated using several standard metrics: IOU (Overlap), Dice coefficient (F-Measurement), Accuracy (Acc), Number of parameters and Number of Epochs needed:

(9) $$\mathrm{Dice}:\;DC=\frac{2\times TP}{2\times TP+FP+FN}$$

(10) $$\mathrm{Jaccard}:\;JC=\frac{TP}{TP+FP+FN}$$

(11) $$\mathrm{Accuracy}:\;Acc=\frac{TP+TN}{TP+TN+FP+FN}$$

where TP, TN, FP and FN are true positive, true negative, false positive and false negative, respectively.

Our EffUnet method is computationally less complex than the ResNet algorithm (see Number of Parameters and Training Time, Table 1). The ResNet algorithm requires 1.134 and 1.93 times more parameters to be tuned (see Ratio, Table 1). EffUnet is also more accurate for detecting boundaries of cup and disc (see IOU, Dice and Accuracy in Table 1) than ResNet.

The EffUnet algorithm achieves high accuracy in detecting the boundaries of the optic disc when compared to 18 published algorithms (Table 2). It achieves the highest *DC* of 0.9991 and the highest *JC* of 0.9983. Its accuracy is very high at *Acc* = 0.9985, which is only 0.0004 smaller than that of the fully convolutional DenseNet, which used the same ORIGA dataset and same train–test split. The rest of the 15 algorithms used other datasets.

The EffUnet algorithm achieved high accuracy in detecting the boundaries of the optic cup when compared to five published algorithms (Table 3). It achieved DC 0.8706, JC 0.7815 and Acc 0.9983. The values of DC and JC are higher than those of DenseNet and the value of Acc was similar to that derived from DenseNet, which also used the ORIGA dataset with the same split to train and test subsets.

The EffUnet algorithm, when trained on ORIGA and fine-tuned on DRISHTI-A, achieves high accuracy in detecting the optic cup and optic disc in DRISHTI-B compared to four published algorithms (Table 4). The model achieves a cup DC 0.9229, cup JC 0.8612, disc DC 0.9991 and disc JC 0.9983, which is the state-of-the-art performance on the DRISHTI-B set.

### 3.2. Segmentation Model: Reliability of Vertical CDR

**Figure 6:**
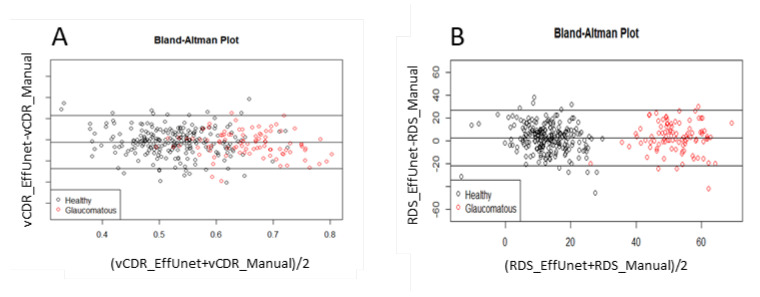
Reliability analysis of (**A**) vertical cup-to-disc ratio (CDR) and (**B**) rim deformation score (RDS) via Bland–Altman plot. Data used: segmentation trained on ORIGA-A, test set is ORIGA-B.

The segmentation model has very good reliability for determining the vertical CDR (vCDR, Figure 6). After EffUnet segmented the cup and disc, the vertical heights of the cup and disc were calculated (in pixels), and the vertical cup-to-disc ratio was calculated (see vCDR_EffUnet in Figure 6). This was then compared to the values from the manual annotation of the images where an ophthalmologist clicks several pixels of cup and disc (see vCDR_Manual in Figure 6, which is the same as vCDR in Figure 1). For this reliability analysis, we used a Bland–Altman analysis (Figure 6A).

### 3.3. EffUnet-SpaGen: Reliability of RDS

The segmentation model has very high reliability in terms of the Rim Deformation Score (RDS; Equation (7); Figure 6B). The RDS values calculated from EffUnet (see RDS_EffUnet, Figure 6B) are in good agreement with those calculated using the manually segmented cup and disc (see RDS_Manual in Figure 6B).

### 3.4. EffUnet-SpaGen: Internal Validation for Glaucoma Detection in ORIGA and DRISHTI Datasets

The accuracy of EffUnet-SpaGen is high in internal validation. We trained both stages of EffUnet-SpaGen on the ORIGA-A data and achieved 0.997 AUROC (Table 5). The CDAR alone gives 0.844 and 0.856 accuracy for ORIGA and DRISHTI, respectively. CDAR improves the accuracy from 0.939 to 0.994 for ORIGA, and 0.879 to 0.923 for DRISHTI, if one variance parameter is used. CDAR improves the accuracy from 0.965 to 0.997 for ORIGA, and 0.923 to 0.969 for DRISHTI, if two variance parameters are used. Therefore, in summary, it improves the accuracy by 3.7 to 5.5%.

### 3.5. Comparison Results of Our Method for ORIGA Dataset

Our approach, EffUnet-SpaGen, on the ORIGA dataset has the best performance published to date (AUROC = 0.997) when compared to state-of-art architectures (Table 6). The Gabor [56] and Wavelet [57] methods use manual features with Support Vector Machine (SVM) classifiers to obtain the diagnostic results. GRI [58] is a probabilistic two-stage classification method to extract the Glaucoma Risk Index. The Superpixel [59] method segments the optic disc and optic cup using superpixel classification for glaucoma screening. Chen et al. [60] and Zhao et al. [61] proposed two convolutional neural network (CNN) methods, both of which achieved good accuracy. MacCormick et al. [5] used dense fully convolutional deep learning (DL) models for segmentation, and a spatial model for Disc Deformation Index (DDI) and classification had high accuracy (0.996 AUROC), but this process was highly computationally intensive (Table 6).

**Figure 7:**
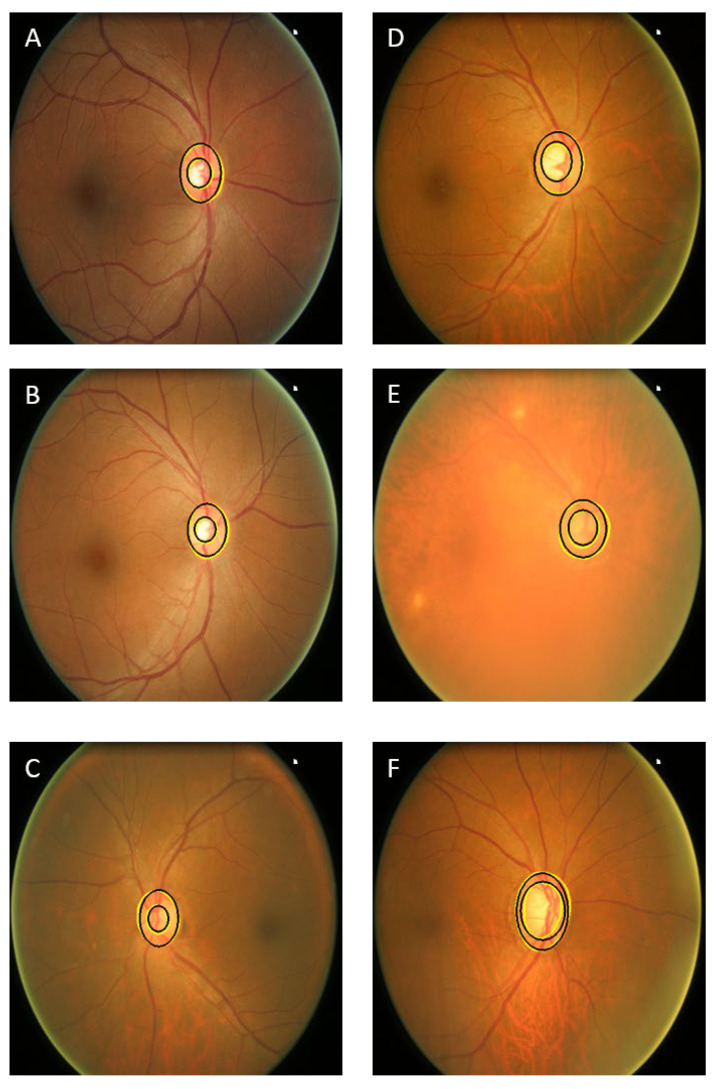
Visual results of several images of normal eyes (**A**–**C**) and glaucomatous eyes (**D**–**F**). The challenging images are (**E**,**C**,**F**). The black contours show the manual annotation and yellow contours show the results of our segmentation approach.

The visual results of our segmentation demonstrate very good results on challenging images compared to manual annotation (Figure 7), including images of poor quality, cases where the blood vessels obscure large parts of the optic cup, images showing very low contrast in the optic disc area and cases of a varying cup and rim size.

## 4. Discussion

We present a new interpretable approach to glaucoma diagnosis, which combines a computationally lean cup and disc segmentation algorithm (EffUnet) with an improved generative spatial algorithm (SpaGen). This hybrid approach is an important improvement over existing machine learning algorithms, allowing for an interpretable explanation of the findings by providing visualization measurements of the cup and disc, on which the diagnosis is based. Additionally, it allows us to present these areas and the key points of interest, such as rim thinning. This approach provides us with a point at which errors can be detected and mitigated, which direct deep learning approaches cannot currently do. Our approach allows lean computation, excellent results with less data, and the incorporation of additional information.

The EffUnet-SpaGen algorithm for the automated grading of optic nerve head images from fundus photographs achieves excellent performance in identifying eyes with glaucoma and distinguishing them from eyes without glaucoma. We have also demonstrated the generalizability of our work to two distinct populations by updating our method for and evaluating it on the DRISHTI dataset. As with all projects in medical imaging, it would be beneficial to demonstrate that these improved results persist in additional datasets and particularly on additional populations. It was demonstrated already that deep learning models for glaucoma, as well as other diseases, experience a drop in performance when evaluated on new populations, even though the imaging may appear to be similar [63]. While we have tested on multiple populations in this work, it is important to continue to evaluate the widest possible demographic. This highlights the need for the development of more publicly available datasets with glaucoma ground truth. To address this issue, we are currently developing segmentation masks for the LAG [64] dataset with Aravind Eye Hospital, Pondicherry, India, in an attempt to alleviate this problem.

In the task of accurately diagnosing glaucoma, we achieved an AUROC of 0.997 on the ORIGA dataset and 0.969 on DRISHTI, performing similarly or better than competing approaches, including [5] (0.996) and [62] (0.88). This represents an almost perfect result for internal validation and is the best performance reported to date for AI algorithms targeted at the diagnosis of glaucoma, compared with results that are publicly available and tested on curated datasets. Furthermore, our AUROC improves on that of a recent deep learning algorithm, which achieved 0.986 [3]. We have also demonstrated that our cup and disc segmentation technique achieves excellent performance compared with previous work.

Both EffUnet and SpaGen are computationally lean, with EffUnet requiring almost half the number of parameters of ResNet34. This allows it to estimate the glaucoma score in less than a second, making our computational speed comparable with Deep Learning approaches while achieving similar results. Furthermore, the interpretation of the results is intuitive: the deformation of the rim is calculated along the whole cup and disc as a deviation from the normal ellipsoid-like shape, meaning that the exact deformation can be easily visualized by a clinician. Our approach also allows us to intuitively factor in additional information such as the cup to disc size and area ratio, which, as we have demonstrated, allows for more accurate results.

## 5. Conclusions

We have presented a supervised hybrid machine and statistical learning classification framework for glaucoma detection from fundus images that are computationally flexible for wide clinical use. We achieved this by introducing a two-step framework consisting of computationally lean automated segmentation (EffUnet) and statistical learning spatial generative algorithm (SpaGen).

The segmentation produced by our proposed AI acts as a device-independent representation of the shape of the cup and disc, up to changes in the field of view and aspect ratio, which our SpaGen algorithm can accommodate. This means that, while we may need to update the segmentation training with new data, we do not need to retrain the glaucoma classification rule.

On the standard benchmark dataset, EffUnet-SpaGen outperformed state-of-art deep-learning methods (0.997 AUROC) while requiring smaller datasets (n = 325) for training the segmentation and classification approaches.

EffUnet is computationally less demanding (using 1.9× fewer parameters than other machine learning approaches), and SpaGen is a generative model that efficiently models the noise in data, requiring only 15 parameters. The 15-parameter model is a probabilistic generative model that efficiently models the ellipsoid shape of the optic nerve head. It shows that there is large data redundancy in the fundus image, with most of the necessary information appearing to lie in the boundaries of the optic nerve head. Combined, this allows EffUnet-SpaGen to be trained efficiently on an n = 325 dataset, which is consistent with a 300-fold decrease in training data compared to [23].

Our work removes the barriers to wider clinical use without requiring a prohibitive amount of training data in a real-world setting. Given it is tested in real clinical settings, this AI will translate to improvements in the management of eye care and help with the prevention of blindness from glaucoma.

## Figures and Tables

**Table 1:** Computational efficiency and accuracy of segmentation of cup and disc jointly via EffUnet and ResNet-Unet. The training dataset is ORIGA-A, the test set is ORIGA-B. Ratio of parameters is the ratio of the number of parameters in a method divided by the number of parameters in the EffUnet method.

Methods	JC	DC	Acc	Number of Parameters	Ratio of Parameters	Training Time (Minutes)
ResNet34-Unet [7]	0.845	0.910	0.9966	24,456,444	1.93	55
ResNet18-Unet	0.846	0.911	0.9967	14,340,860	1.134	49
EffUnet (our method)	0.854	0.916	0.9968	12,641,459	1	42

**Table 2:** Comparison of segmentation methods for optic disc. Note: [31,32,33] performed segmentations of both cup and disc.

Author	Method	Optic Disc			Dataset
		DC	JC	Acc	
Wong et al. [34]	Support vector machine-based classification mechanism	-	0.940	0.990	SiMES
Yu et al. [30]	Directional matched filtering and level sets	-	0.844	-	MESSIDOR
Mookiah et al. [35]	Attanassov intuitionistic fuzzy histon (A-IFSH) based method	0.920	-	0.934	Private
Giachetti et al. [36]	Iteratively refined model based on contour search constrained by vessel density	-	0.861	-	MESSIDOR
Dashtbozorg et al. [37]	Sliding band filter	-	0.890	-	MESSIDOR
		-	0.850	-	INSPIRE-AVR
Basit and Fraz [38]	Morphological operations, smoothing filters, 3* and the marker controlled watershed transform	-	0.710	-	Shifa
		-	0.456	-	3*CHASE-DB1
		-	0.547	-	3*DIARETDB1
		-	0.619	-	DRIVE
Wang et al. [39]	Level set method	-	0.882	-	DRIVE
		-	0.882	-	DIARETDB1
		-	0.891	-	DIARETDB0
Hamednejad et al. [40]	DBSCAN clustering algorithm	-	-	0.782	DRIVE
Roychowdhury et al. [41]	Region-based features and supervised classification	-	0.807	0.991	DRIVE
		-	0.802	0.996	DIARETDB1
		-	0.776	0.996	DIARETDB0
		-	0.808	0.991	CHASE-DB1
		-	0.837	0.996	MESSIDOR
		-	0.729	0.985	STARE
Girard et al. [42]	Local K-means clustering	-	0.900	-	MESSIDOR
Akyol et al. [43]	Keypoint detection, texture analysis, and visual dictionary	-	-	0.944	DIARETDB1
		-	-	0.950	DRIVE
		-	-	0.900	ROC
Abdullah et al. [44]	Circular Hough transform and grow-cut algorithm	-	0.786	-	DRIVE
		-	0.851	-	DIARETDB1
		-	0.832	-	CHASE-DB1
		-	0.879	-	MESSIDOR
		-	0.861	-	Private
Tan et al. [45]	7-Layer CNN	-	-	-	DRIVE
Zahoor et al. [46]	Polar transform	-	0.874	-	DIARETDB1
		-	0.844	-	MESSIDOR
		-	0.756	-	DRIVE
Sigut et al. [47]	Contrast based circular approximation	-	0.890	-	MESSIDOR
Noor et al. [31]	Colour multi-thresholding segmentation	0.590	-	0.709	DRIVE
Khalid et al. [32]	Fuzzy c-Means (FCM) and morphological operations	-	-	0.937	DRIVE
Yin et al. [48]	Statistical model	-	0.920	-	ORIGA
Fu et al. [14]	Multi-label deep learning and Polar transformation (DL)	-	0.929	-	ORIGA
Al-Bander et al. [33]	Fully convolutional DenseNet	0.965	0.933	0.999	ORIGA
Proposed method	EffUnet	0.999	0.998	0.999	ORIGA

**Table 3:** Comparison of segmentation methods for optic cup.

Author	Method	Optic Cup			Dataset
		DC	JC	Acc	
Hatanaka et al. [49]	Detection of blood vessel bends and features determined from the density gradient	-	-	-	Private
Almazroa et al. [50]	Thresholding using type-II Fuzzy method	-	-	0.761	BinRushed
		-	-	0.724	Magrabi
		-	-	0.815	MESSIDOR
Noor et al. [31]	Colour multi-thresholding segmentation	0.510	-	0.673	DRIVE
Khalid et al. [32]	Fuzzy c-Means (FCM) and morphological operations	-	-	0.903	DRIVE
Yin et al. [51]	Sector-based and intensity with shape constraints	0.830	-	-	ORIGA
Yin et al. [48]	Statistical model	0.810	-	-	ORIGA
Xu et al. [52]	Low-rank superpixel representation	-	0.744	-	ORIGA
Tan et al. [53]	Multi-scale superpixel classification	-	0.752	-	ORIGA
Fu et al. [14]	Multi-label deep learning and Polar transformation	-	0.770	-	ORIGA
Al-Bander et al. [33]	Fully convolutional DenseNet	0.866	0.769	0.999	ORIGA
Proposed method	EffUnet	0.870	0.782	0.998	ORIGA

**Table 4:** Comparison of segmentation methods for optic cup and disc. The model was finetuned on DRISHTI-A (n = 50 images) and evaluated on DRISHTI-B set (n = 51 images).

Author	Optic Disc		Optic Cup	
	DC	JC	DC	JC
Sevastopolsky [54]	-	-	0.850	0.750
Zilly et al. [55]	0.973	0.914	0.871	0.850
Al-Bander et al. [33]	0.949	0.904	0.828	0.711
Shuang et al. [7]	0.974	0.949	0.888	0.804
Proposed method	0.999	0.998	0.923	0.861

**Table 5:** Ablation study of accuracy of EffUnet-SpaGen in internal validation on ORIGA and on DRISHTI. For ORIGA: train set for segmentation and glaucoma detection is ORIGA-A (n = 325) (253:72 of healthy: glaucomatous), test set is ORIGA-B (n = 325) (229:96 of healthy: glaucomatous). For DRISHTI: train set for segmentation is whole ORIGA and DRISHTI-A, train set for glaucoma detection is ORIGA and test is DRISTHI-B. CDAR is the Cup/Disc Area Ratio.

Segmentation Model	Generative Model (n of Parameters)	Results for ORIGA (Top), DRISHTI (Bottom)				
		AUROC	Sen	Spe	PPV	NPV
EffUnet	CDAR (2)	0.844	0.847	0.726	0.882	0.663
		0.856	0.737	0.923	0.966	0.545
EffUnet	CDR profile of 24values and 1 variance parameter (13)	0.939	0.842	0.921	0.816	0.934
		0.879	0.789	0.923	0.968	0.600
EffUnet	CDR profile of 24 values and 2 variance parameters (14)	0.965	0.863	0.961	0.901	0.944
		0.933	0.895	0.923	0.971	0.750
EffUnet	CDR profile of 24 values and 1 variance parameters and CDAR (14)	0.994	0.979	0.961	0.912	0.991
		0.923	0.842	0.923	0.970	0.667
EffUnet	CDR profile of 24 values and 2 variance parameters and CDAR (15)	0.997	0.989	0.974	0.940	0.996
		0.969	0.947	0.923	0.973	0.857

**Table 6:** Detection of glaucoma in ORIGA. The training set is ORIGA-A and the test set is ORIGA-B.

Author	Method of Glaucoma Detection	AUROC
Dua et al. [57]	Wavelet	0.660
Acharya et al. [56]	Gabor	0.660
Cheng et al. [59]	Superpixel	0.830
Bock et al. [58]	GRI	0.810
Chen et al. [60]	CNN	0.830
Zhao et al. [61]	CNN	0.869
Liao et al. [62]	EAMNet	0.880
MacCormick et al. [5]	DL + DDI	0.996
Proposed method	EffUnet-SpaGen	0.997

## Data Availability

The ORIGA dataset [29] is a publicly available subset of the data from the Singapore Malay Eye Study, collected from 2004 to 2007 by the Singapore Eye Research Institute and funded by the National Medical Research Council. All images were anonymized before release. DRISHTI -GS1 [30,65] is a dataset collected and annotated by Aravind Eye Hospital, Madurai, India. Both datasets are publicly available and can be accessed by contacting the authors of the corresponding manuscripts.
